# More Frequent Internet Use during the COVID-19 Pandemic Associates with Enhanced Quality of Life and Lower Depression Scores in Middle-Aged and Older Adults

**DOI:** 10.3390/healthcare9040393

**Published:** 2021-04-01

**Authors:** Anna-Stiina Wallinheimo, Simon L. Evans

**Affiliations:** Faculty of Health and Medical Sciences, University of Surrey, Guildford, Surrey GU2 7XH, UK; anna-stiina.wallinheimo@surrey.ac.uk

**Keywords:** older adults, coronavirus, internet, mental health, depression symptoms, quality of life, loneliness, communication, social distancing, digital divide

## Abstract

Concerns have been raised regarding middle-aged and older adults’ mental health during the coronavirus outbreak. The aim of the current study was to characterise associations between internet use (frequency and purpose), depression symptoms and Quality of Life (QoL) during the pandemic, in individuals aged 55–75. Data (N = 3491) were drawn from the English longitudinal study of ageing (ELSA) cohort study collected in June/July 2020 (while social distancing measures were in place). Associations with frequency of use were tested using analysis of covariance (ANCOVAS), controlling for covariates such as wealth and education. Type of internet use (for communication, information search) was also analysed amongst frequent users. Significant effects of frequency of use were observed (*p* = 0.01 for depression, *p* < 0.001 for QoL), with lower depression symptoms and higher QoL scores amongst more frequent users. Regarding purpose of use, those who reported using the internet for communication purposes had higher QoL. However, use for health-related or Government services information searching was associated with more depression symptoms. Results provide important information regarding the potential benefits of internet use for middle-aged and older people, suggesting that strategies to increase internet usage (particularly for communication) might benefit middle-aged and older adults’ mental health and counter isolation as the coronavirus crisis continues to evolve.

## 1. Introduction

The coronavirus (COVID-19) pandemic has had profound psychological and social consequences on populations globally. All age groups have been affected, but middle-aged and older people are at significantly higher risk of negative health outcomes and mortality if they contract the virus. Moreover, concerns have been raised regarding middle-aged and older adults’ mental health, given that loneliness and isolation would be exacerbated as lockdown measures were implemented. Social participation has a well-documented association with mental health and Quality of Life (QoL) in older adults [1]. In the UK, the first nationwide “lockdown” was imposed in March 2020: all unnecessary social contact ceased and non-essential businesses closed. Individuals were ordered to remain at home except to purchase essentials. Under lockdown, the closing of community organisations and limits on visits from family members will have undoubtedly impacted levels of social participation amongst middle-aged and older people. A review of studies into the impact of COVID-19 lockdowns on mental health in older adults [2] found that of eight cross-sectional studies reviewed, six reported negative effects including higher depression and loneliness. Further, a greater reduction in social contacts due to lockdown measures has been associated with higher mental health complaints [3]. While there is some evidence to suggest that older adults have proven more resilient than initially feared [4], investigations into the effects of lockdown and social isolation on mental health should be prioritised [5], to inform strategies that promote wellbeing as the situation evolves and further lockdowns may be required. 

Holmes and O’Connor [5] highlight that issues around isolation and mental health in older adults might be exacerbated by the “digital divide”, whereby older people make less use of information and communication technologies, for reasons including lack of skills, confidence and accessibility issues. Survey data collected during the early months of the pandemic indicated that people with higher levels of internet competency were more likely to increase their digital communication activities during the pandemic [6]. Although internet use and competency is becoming more prevalent amongst UK adults aged 50 years and older, it is still markedly lower when compared to younger adults [7]; providing relevant training programmes could have broad benefits for older adults [8]. As a means of mitigating the negative effects of lockdown on the mental health of middle-aged and older people, internet use could be an important factor. However, data regarding associations between internet use, mental health, and QoL amongst middle-aged and older people under lockdown are lacking.

There is good evidence that internet use is a potential protective factor against poor mental health in older adults. A recent review considered 18 quantitative cross-sectional studies and found that all studies but 1 reported statistically significant correlations between internet use and mental health in later life [9]. More recent studies confirm such associations [10]. Longitudinally, cotton and Ford [11] found internet use to reduce the probability of subsequent depression amongst retired US adults by a third; the effects were largest for people living alone, supporting the notion that internet use might reduce depression risk by mitigating social isolation and loneliness. Using the internet for communication may enhance a sense of connectedness with others, and studies have shown that a higher frequency of going online is associated with lower loneliness scores in older adults, measured cross-sectionally and controlling for number of friends and family [12]. Consistently with this, Sum and Mathews [13] found that older adults who used the internet as a communication tool had lower levels of loneliness, and better well-being. Another recent, critical, review [14] focused on use of the internet and QoL, concluding that the majority of studies found positive effects of internet use by older adults on QoL, particularly by enhancing communication with family and friends.

The English longitudinal study of ageing (ELSA) is a longitudinal survey of people aged 50 and over in England [15]. Every two years, it collects data across a wide variety of topics including lifestyle behaviours, health and wellbeing. Questions pertaining to internet use are included, although these have varied across waves. Coding internet use as a dichotomous variable(yes/no), Quintana and Cervantes [16] found that ELSA participants who were internet users reported higher satisfaction with life. Lam and Jivraj [17] analysed more detailed data from ELSA gathered in the more recent waves 6–8 (2012–2017) to examine the relationship between internet use (both frequency and purpose) and two mental health outcomes (depression symptoms, life satisfaction). Deteriorating life satisfaction was more likely amongst infrequent internet users (defined as monthly or less) compared to frequent (daily) users; education and occupational class had moderating effects. Frequency of use did not predict depressive symptoms. However, in terms of type of use, those who used the internet for communication reported a lower depression score and better life satisfaction, while those using the internet for information access reported lower life satisfaction scores. 

ELSA conducted a COVID-19 sub-study in June/July 2020, gathering data on various lifestyle and health measures so as to investigate the effects of the COVID-19 crisis on middle-aged and older people in England. As outlined above, the evidence suggests that the COVID-19 pandemic and ensuing social restrictions have had significant effects on the mental health of older people, with effects on isolation and loneliness a factor in this. Previous (pre-pandemic) studies have identified internet use (particularly for communication) as being beneficial for QoL, depression, and loneliness in older people, but little is known about the nature of these relationships during the unprecedented social restrictions imposed during the COVID outbreak. Based on prior evidence, one might predict the benefits of frequent internet use to be enhanced during the COVID crisis, with internet use potentially mitigating the deleterious consequences of social isolation for middle-aged and older people’s mental health. On the other hand, there is evidence that opposing effects might be possible. A cross-sectional study among Chinese citizens aged 18–85 in Feb 2020 found that more frequent social media use during the pandemic correlated with higher anxiety and depression symptoms [18]. This might be due to the high levels of COVID-19 information being transmitted via social media channels, exacerbated by disinformation and false reports about the virus. Further, negative sentiments triggered by this (such as fear and anxiety) could be contagious across social media networks [19]. Reinforcing this, the previous study by Lam and Jivraj [17] found that accessing the internet for information purposes had negative consequences for life satisfaction in older people, even pre-pandemic.

The aim of the current study is therefore to characterise the impact of internet use on depression symptoms and QoL during the pandemic amongst individuals aged 55–75, drawn from the ELSA cohort sub-study in June/July 2020. Importantly, we considered both self-reported frequency of use and purpose. Based on previous findings, we hypothesised positive effects of frequency of use on depression symptoms and QoL. Regarding purpose, given the evidence outlined above, we focused just on communication (voice/video calls) and information purposes, and hypothesised the positive effects of use for communication, and negative effects of use for information purposes. Relevant covariates (including household wealth and education) were included in the analyses. 

## 2. Materials and Methods

### 2.1. Data and Sample

Data are drawn from the English longitudinal study of ageing (ELSA) cohort study, which has been described elsewhere [15]. Data are publicly available at https://beta.ukdataservice.ac.uk/datacatalogue/series/series?id=200011. The data were accessed under project number 206540 in Jan 2021. ELSA collects data from a representative sample of adults aged 50 years and over living in private households in England, every 2 years. Additionally, in June/July 2020, ELSA conducted a COVID-19 sub-study to investigate the effects of the COVID-19 crisis on middle-aged and older people in England. We used this sub-study wave of data collection to address the study aims. The data collection comprised a combination of internet and telephone assessments. The ELSA sample members were contacted by post to take part in an online survey of 30 min. There was a GBP 10 incentive to participate. Those who were unable to participate online were contacted by the NatCen (National Centre for Social Research) telephone unit to carry out a computer-assisted telephone interview (CATI) over a time period of 3–4 weeks. ELSA was conducted in accordance with the Declaration of Helsinki. Ethical approval was granted by the Multicentre Research and Ethics Committee (MREC 01/2/91). Participants gave informed consent to take part. For this study the following inclusion criteria were applied: aged 55–75, currently living in a private household (i.e., not hospitalised/in care). We excluded those aged above 75, so as to minimise potential confounds related to age-related health issues, as full health data were not available. All data were drawn from the COVID-19 sub-study wave (June/July 2020), apart from education and net household wealth, which were drawn from the ELSA wave 9 conducted in June 2018–May 2019. Only participants with provided data on all the variables under study here were included. This meant that we excluded 1049 participants due to missing data (e.g., wealth quintile), yielding a final sample of 3491. The excluded participants included those who did not complete wave 9 of ELSA; as such, there was no wealth quintile information available for these participants.

### 2.2. Depression and Quality of Life (QoL)

Depressive symptoms were measured using the seven-item Center for Epidemiologic Studies depression scale (CES-D) short form (CES-D-SF). The CES-D-SF asks about the presence of the following symptoms in the previous week: depression, lack of happiness, loneliness, sadness, that everything was an effort, sleep was restless, inability to get going, and lack of energy. Each question is responded to as either yes or no, summing to a total score that ranges from 0 to 7, with higher scores indicating more depressive symptoms. The CES-D-SF has comparable psychometric properties to the CES-D, has good internal and test–retest reliability, and a total score of ≥ 3 on the CES-D-SF corresponds with an interview-based clinical diagnosis of depression [20]. Quality of life (QoL) was measured using the 12-item control, autonomy, self-realization and pleasure scale (CASP-12). Each item was rated on a 4-point Likert scale (1–4) totalling to scores ranging from 12 to 48, with higher scores indicating higher QoL. Validity has been established: the Cronbach’s alpha is in the range 0.74–0.792 [21]. 

### 2.3. Frequency and Purpose of Internet Use

Frequency and purpose of internet use was assessed by self-report. For frequency, the question asked was “Since the coronavirus outbreak, on average, how often did you use the Internet or email?” Six internet use frequency options were included, and we collapsed these to form four: (1) more than once a day, (2) once a day, (3) once a week, and (4) less than once a week. Category (4) comprised the 3 original categories “At least once a month (but not every week)/Less than monthly/Never. For our analyses on effects of type of internet use, we conducted these within “Frequent” internet users only, who we defined as individuals who used the internet more than once a day or once a day (combining categories 1 and 2). 

For the purpose of internet use, the question asked was “For which of the following activities did you use the Internet in the last 3 months?” There were ten options given: (1) Sending/receiving e-mails; (2) Making video calls or voice calls (using applications such as Skype, WhatsApp, or FaceTime); (3) Finding information on health-related issues; (4) Managing my finances (online banking, paying bills, and paying taxes); (5) Shopping/buying goods or services; (6) Using social networking sites (Facebook, Twitter, LinkedIn, Instagram, blogging, or Flickr); (7) Reading news/newspaper/blog websites; (8) Streaming TV/videos/radio (BBC iPlayer, Netflix, Amazon Prime, YouTube), listening to music (Spotify, Apple Music), playing online games, or reading eBooks; (9) Getting information about Government services (benefits, taxes, a driving licence, or passport, etc.); and (10) None of the above. Participants responded yes or no to each option and they could indicate more than one answer.

### 2.4. Other Internet Use-Related Questions

The participants were asked additional questions about their current internet use. The first question was “Since the coronavirus outbreak started would you say your use of the internet is.” (1) Less than it was; (2) About the same; (3) More than it was. The participants were then asked “Would you like to be able to use the internet more frequently or for more things?” (1) Yes; (2) No. Those that answered ‘Yes’ were then asked: “There are a number of reasons why people do not use the internet more. Which of the following apply to you?” (1) My IT skills are not good enough; (2) I do not trust the internet (fraud, sharing personal data); (3) I do not have access to good enough equipment; (4) I do not have good enough access to broadband; (5) My vision is not good enough to use the equipment; (6) Health problems (excluding vision problems) stop me from using the equipment; (7) I have no reason to use it more; (8) It takes too much time; and (9) Another reason (please specify). The participants indicated their answer with a yes or no response (participants could indicate more than one answer).

### 2.5. Covariates

We included age, gender, net household wealth (total net nonpension household wealth: financial, physical, and housing wealth, minus debts, coded by quintile, calculated by the ELSA study team), current employment status (coded as: retired; employed, paid/unpaid leave; self-employed; self-employed, not working; unemployed; sick/disabled; and looking after home), the number of people in the household (coded as: “living alone” or “not alone”) and education (seven levels, from Degree level through to “none”) as covariates in all the statistical analyses, to control for their effects on the dependent variables. 

### 2.6. Data Analysis Plan

To investigate the effects of frequency of internet use, a one-way between-groups analysis of covariance (ANCOVA) was conducted with frequency of internet use (more than once a day, every day, once a week, or less than once a week) as the between-groups factor. Two separate ANCOVAs were conducted, with depression (CES-D-SF) and QoL (CASP-12) as dependent variables. To investigate the effects of type of internet use, we conducted a separate analysis within “frequent” (once a day or more) internet users only. We considered three types of use: (1) Communication (Making video calls or voice calls); (2) Finding information on health-related issues; (3) Getting information about Government services. Amongst frequent users, separate ANCOVAS compared those who reported using the internet for the given use against those who did not, with depression (CES-D-SF) and QoL (CASP-12) as dependent variables. The significance threshold was set at *p* > 0.05 for all analyses. Assumptions for ANCOVAs were met: the data was normally distributed (Shapiro–Wilk’s test *p* > 0.05 for all variables); the assumption of equality of error variances was met as Levene’s test was non-significant for all the comparisons conducted.

## 3. Results

### 3.1. Descriptive Statistics

The data comprised of 3491 participants (age: *M* = 67.18, *SD* = 5.23). Females represented 57% of the sample. Table 1 summarises the characteristics of the total sample and the frequency of internet use. During the coronavirus outbreak, 57% reported using the internet more than once a day, 24% used it every day, 7% once a week, and 12% less than once a week. The most common purpose of using the internet was to send and receive e-mails (90%), followed by shopping and buying goods and services (77%), making video or voice calls (65%), and managing finances (63%). In total, 3% of the participants reported that their internet use was less than it was before the coronavirus outbreak, 52% said it was about the same, and 45% confirmed that it was more. Furthermore, 14% of the participants wanted to use internet more and 86% of the participants did not want to use internet more frequently or for more things. The main reasons for people not to use the internet were: the participants did not feel that their IT skills were good enough (47%); the participants did not trust the internet (fraud, sharing personal data) (32%); they did not have access to good enough equipment (10%) or good enough access to broadband (13%); and there was no reason to use it more (25%). Overall, 26% of the participants scored 3 or more on the CES-D-SF (this cut-off indicates a clinical diagnosis of depression [20]). 

### 3.2. Relationships between Frequency of Internet Use and Depression Symptoms/QoL

**Figure 1:**
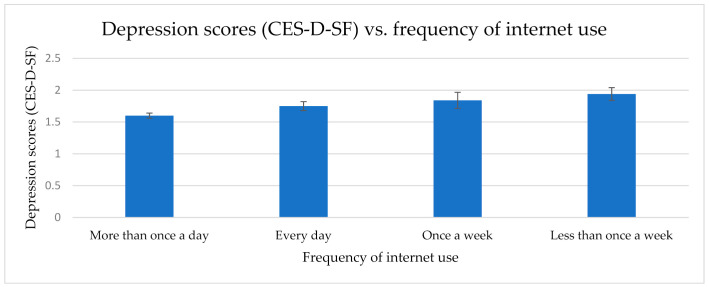
Mean depression scores (w/standard errors) vs. frequency of internet use. A significant main effect of frequency was observed (*p* = 0.01), and there was a significant difference between the most and least frequent users (*p* < 0.001).

A one-way (frequency of internet use: more than once a day, every day, once a week, or less than once a week) between-groups analysis of covariance (ANCOVA) was conducted with depression (CES-D-SF) as the dependent variable, whilst controlling for age, gender, net household wealth, current employment status, the number of people in the household, and education. There was a significant main effect of frequency of internet use, *F*(3, 3481) = 3.81; *p* = 0.01; ŋ_p_^2^ = 0.003. The more frequent internet users had lower depression scores: more than once a day (*M* = 1.60; *SE* = 0.04), every day (*M* = 1.75; *SE* = 0.07), once a week (*M* = 1.84; *SE* = 0.13), and less than once a week (*M* = 1.94; *SE* = 0.10) (Figure 1). Pairwise comparisons (with Bonferroni correction) identified a significant difference between Group 1 (more than once a day) and Group 4 (less than once a week), *p* < 0.001. Similar effects were seen when depression scores were categorised using a cut-off of ≥3 on the CES-D-SF (corresponding with a clinical diagnosis of depression): 23% of the most frequent users met this criterion for a depression diagnosis, compared to 29% of the every day users, 31% of the individuals who used the internet once a week, and 37% of the participants who used the internet less than once a week. 

**Figure 2:**
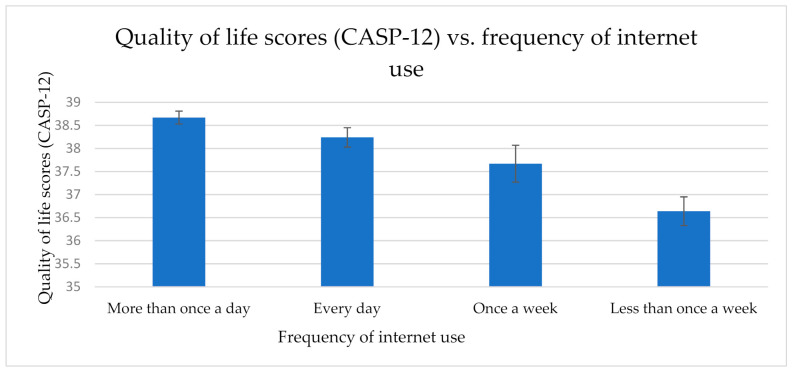
Mean QoL scores (w/standard errors) vs. frequency of internet use. A significant main effect of frequency was observed (*p* < 0.001), there was a significant difference between the “less than once a week” group and the “more than once a day” and “every day” groups (*p* < 0.001).

A one-way (frequency of internet use: more than once a day, every day, once a week, or less than once a week) between-groups analysis of covariance (ANCOVA) was conducted with QoL (CASP-12) as the dependent variable. We controlled for age, gender, net household wealth, current employment status, the number of people in the household, and education. There was a significant main effect of frequency of internet use, *F*(3, 3481) = 11.82; *p* < 0.001; ŋ_p_^2^ = 0.01. More frequent use was associated with higher QoL scores: more than once a day (*M* = 38.67; *SE* = 0.14), every day (*M* = 38.24; *SE* = 0.21), once a week (*M* = 37.67; *SE* = 0.40), and less than once a week (*M* = 36.64; *SE* = 0.31) (Figure 2). The pairwise comparisons (with a Bonferroni correction) identified a significant difference between Group 1 (more than once a day) and Group 4 (less than once a week), *p* < 0.001; and between Group 2 (every day) and Group 4 (less than once a week), *p* < 0.001.

### 3.3. Relationships between Purpose of Internet Use and Depression Symptoms/QoL

To investigate the relationship between the purpose of internet use, depression symptoms and QoL, we conducted analyses amongst frequent internet users only (defined as internet use of once a day or more): this was because we were specifically interested in assessing how type of use might relate to the frequency effects on depression and QoL, and because the “type of use” data are less informative (and potentially less reliable) amongst those who use the internet once a week or less. 

#### 3.3.1. Health-Related Information Search

Two one-way (internet use for health-related information search: yes or no) between-groups analysis of covariance (ANCOVA) were conducted with depression (CES-D-SF) and QoL (CASP-12) as the dependent variables, whilst controlling for age, gender, net household wealth, current employment status, the number of people in the household, and education. There was a significant main effect of internet use for health-related information search on depression, *F*(1, 2820) = 40.71, *p* < 0.001, ŋ_p_^2^ = 0.01. The frequent users who reported this type of use had higher depression scores (*M* = 1.83, *SE* = 0.05) than those who did not (*M* = 1.38, *SE* = 0.05). There was also a significant main effect on QoL scores *F*(1, 2820) = 46.35, *p* <.001, ŋ_p_^2^ = 0.02. The frequent users who reported this type of use had lower QoL scores (*M* = 37.92, *SE* = 0.15) than those who did not (*M* = 39.42, *SE* = 0.16).

#### 3.3.2. Government Services Information Search

Searching for information on Government services had a significant effect on depression scores *F*(1, 2820) = 14.42, *p* < 0.001, ŋ_p_^2^ = 0.005. The frequent users who reported this type of use had higher levels of depression scores (*M* = 1.75, *SE* = 0.05) than those who did not (*M* = 1.48, *SE* = 0.05). There were no significant results of this type of use on QoL scores.

#### 3.3.3. Communication (i.e., Making Video or Voice Calls)

There was a significant main effect of internet use for communication on QoL, *F*(1, 2820) = 5.86, *p* = 0.02, ŋ_p_^2^ = 0.002. The frequent users who reported this type of use had higher QoL scores (*M* = 38.90, *SE* = 0.13) than those who did not (*M* = 38.31, *SE* = 0.20). There were no effects of this type of use on depression scores.

## 4. Discussion

In this study we investigated the relationships between internet use (frequency and purpose) and both depression symptoms and QoL, in a sample of 3491 older adults (aged 55–75) using self-reported data collected in June/July 2020. Previous data, collected before the COVID-19 pandemic, support a positive association between internet use frequency and measures of mental health and life satisfaction, both in the ELSA cohort utilised here [16,17] and other cohorts/populations [9,10,11]. The current study analysed data collected while lockdown restrictions and social distancing measures were in place throughout the UK, to investigate and assess whether these relationships were present (and potentially more significant) under these conditions. The current study provides valuable insight by exploring effects in a large, well-characterised sample, allowing important covariates (e.g., household wealth, education) to be controlled for. Further, well-validated outcome measures were used; this has been noted as a weakness of many previous studies into QoL in particular [14]. Frequency of internet use was seen to be high; when questioned about their internet use during the coronavirus outbreak, 57% reported using the internet more than once a day, and 45% reported using the internet more than before the outbreak. The most common purposes of use were for email, shopping, and making voice/video calls. In total, 14% reported wanting to use the internet more; amongst these, the major reason for not doing so was lack of IT skills, followed by lack of trust (worry about fraud for example).

We predicted a relationship between frequency of use and measures of depression symptoms and QoL. A higher frequency of internet use has previously been linked (pre-pandemic) to lower loneliness scores in older adults [11], and the use of internet for communication has been linked to a lower depression score and better life satisfaction [17]. These facts would suggest that internet use could be highly beneficial for mitigating the deleterious consequences of the COVID-19 social restrictions, and isolation, amongst older people. The current findings support this. We found a clear relationship between frequency of use and depression symptoms and QoL. Those who used the internet more than once a day reported significantly lower depression symptoms and higher QoL, compared to those who used the internet less than once a week, after controlling for a range of relevant covariates including socio-economic status. Specifically, only 23% of individuals who used the internet more than once a day met the criteria for a depression diagnosis, while 37% of those who used it less than once a week met the criteria for depression. In terms of QoL, we saw that those using the internet more than once a day had a mean CASP-12 score of 38.67, while those who used it less than once a week had a mean of 36.64. Although there are not established cut-off criteria for the CASP-12, some studies (e.g., [22]) have used a score of 37 as the boundary between moderate and high QoL based on data from a large cross-European study [21]. This suggests that, in this sample, frequent internet use is associated with “high” QoL, while those who use the internet less than once a week report only “moderate” QoL. 

Although the direction of causality cannot be inferred from the present cross-sectional study, previous longitudinal work has shown that interventions to promote internet use amongst older adults can reduce loneliness and depression [23]. The current study found the effects of frequency of use to be highly significant (*p* < 0.001 for QoL, and *p* = 0.01 for depression). While the majority of previous (pre-pandemic) studies have found similar relationships [9,14], some studies found no effects [24]; other discordant findings have been noted pre-pandemic, particularly for QoL [14]. Thus, the highly significant relationships between frequency of use and both QoL and depression symptoms identified here might be at least partly attributable to the social distancing and lockdown restrictions that were in place, supporting the notion that internet usage might be of particular benefit under these circumstances. 

Follow-up analyses in frequent users (those who used the internet at least once a day) investigated type of use and shed further light on the main findings. Those who used the internet for communication purposes (video/voice calls) had higher QoL than those who did not. This accords with previous studies showing that using the internet to communicate can enhance the well-being of older adults by reducing loneliness [13,23]. Again, this might have been particularly applicable at the time point under study, with using the internet for communication helping to compensate for the social restrictions in place. On the other hand, use for health-related information searching was associated with lower QoL and higher depression symptoms. This is not surprising, as it presumably reflects the usage habits of those individuals with health concerns. The question included in the survey did not differentiate between searching for COVID-related versus other health issues, so COVID-related worry could be a contributor to the lower QoL and higher depression symptoms seen amongst those who used the internet for this purpose. Indeed, a study using data from the USA showed that a significant spike in COVID-related information-seeking occurs when a local case is announced [25]. Previous work in the ELSA cohort also found that those who used the internet for information access reported lower life satisfaction scores [17]. It should be noted that in the ELSA sub-study wave of data collection analysed here, the categories of use differed from previous waves, so direct comparison was not possible. We also found that those who reported searching for Government services information had significantly higher depression scores; again this could reflect searches for information on COVID-related community-level policies (e.g., quarantine, school closures, testing), thus indicating greater COVID-related worry. In contrast, some previous studies have found positive relationships of QoL with information search, with Karavidas and Lim [26] reporting that this was due to various factors, including a greater feeling of independence due to enhanced access to information (including health-related information). This supports the suggestion that COVID-related concerns might be an explanatory factor in the negative relationships identified at the time point studied here. The lack of specificity in the questions did not allow us to explore this further, but could be the subject of future work. This is an important limitation of the current study. Additionally, given the exclusion criteria employed here (maximum age 75 and the exclusion of those not living in private residences), further work is needed to determine whether findings are generalisable to older age groups and those in care. Additionally, the lack of full health data on participants is a limitation. We set an upper age limit of 75 to minimize the confounding influence of age-related health issues. However, this approach cannot fully account for such confounders. Other important study limitations that should be noted include the cross-sectional design, and the reliance on self-report data. Further work examining frequency and usage patterns longitudinally would be beneficial. A finer categorization of use (e.g., accessing of COVID-related internet content) would be beneficial, as would the inclusion of objective measures. However, the analyses of usage purposes conducted here still add important details to the main overall finding that greater internet use links to lower depression symptoms and higher QoL amongst older people. 

In sum, this study in middle-aged and older adults found highly significant relationships between internet use frequency during the COVID crisis and measures of depression symptoms and QoL. Use for communication purposes was associated with higher QoL, while use for information searching was linked to lower QoL and higher depression symptoms. These results support the suggestion that promoting internet use, for communication in particular, could be beneficial for mental health and QoL amongst middle-aged and older people as the crisis continues to unfold, by alleviating feelings of isolation due to social restrictions. However, the results regarding information-searching also highlight the potential downsides of internet use. This accords with a recent study [27] showing generally increased use of social media in 2020, which for some served as a coping mechanism to combat isolation, but caused deleterious effects on mental health in others: the role of greater exposure to crisis-related news was important in explaining this. Thus, while the current findings support the suggestion that addressing the “digital divide” and implementing strategies that facilitate internet use amongst older people might benefit mental health and counter isolation during the crisis [5], some forms of information access might not be beneficial. Crisis-related news transmitted via news networks and social media channels, alongside disinformation and false reports about the virus, could provoke anxiety [18], and this must be considered in any intervention plan to promote wellbeing by facilitating internet access amongst older people. 

## Figures and Tables

**Table 1:** Summary of participant characteristics by frequency of internet use (categories 1–4).

Internet Usage:	More Than Once a Day	Once a Day	Once a Week	Less than Once a Week	Total
**QoL (CASP-12)**					
Mean (SD)	38.99 (5.94)	38.04 (6.24)	37.26 (6.62)	35.75 (7.08)	38.25 (6.30)
Participants, N	2000	828	232	431	3491
**Depression (CES-D-SF)**					
Mean (SD)	1.51 (1.86)	1.80 (2.09)	1.96 (2.22)	2.20 (2.26)	1.69 (2.01)
Clinical depression	23%	30%	31%	37%	26%
Participants, N	2000	828	232	431	3491
**Age (years)**					
Mean (SD)	66.46 (5.67)	67.56 (5.32)	68.14 (5.25)	69.31 (4.62)	67.18 (5.23)
Participants, N	2000	828	232	431	3491
**Gender**					
Female, n (%)	54%	59%	67%	61%	57%
Participants, N	2000	828	232	431	3491
**Net household wealth**					
Wealth quintile (1–5)	3.60 (1.36)	3.24 (1.35)	3.06 (1.36)	2.74 (1.39)	3.37 (1.39)
Participants, N	2000	828	232	431	3491
**The number of people living in the household**					
Living alone	18%	23%	23%	34%	22%
Participants, N	2000	828	232	431	3491
**Current employment**					
Retired	63%	65%	69%	75%	65%
Employed	24%	18%	14%	9%	20%
Other	13%	17%	17%	16%	15%
Participants, N	2000	828	232	431	3491
**Education**					
Degree level	35%	17%	10%	3%	25%
Higher education	19%	17%	16%	8%	17%
Secondary or lower	58%	46%	66%	74%	89%
Participants, N	2000	828	232	431	3491
**Purpose of internet use**					
Sending e-mails (1)	96%	84%	68%	55%	90%
Making video calls (2)	75%	53%	35%	27%	65%
Finding health-r. (3)	51%	38%	27%	12%	45%
Managing finances (4)	73%	50%	31%	18%	63%
Shopping (5)	85%	68%	46%	37%	77%
Social networking (6)	59%	43%	22%	20%	51%
Reading news (7)	67%	48%	26%	23%	58%
Streaming (8)	59%	38%	22%	25%	50%
Info on Government (9)	48%	31%	19%	18%	41%
Participants, N	2000	828	232	431	3491

Note. Clinical depression, scores of 3 or more out of 7 on the CES-D-SF. Wealth quintile (1 = least affluent). Current employment (9 levels, summarised here as: Employed (includes self-employed), Retired, or Other (paid/unpaid leave; self-employed, not working; unemployed; sick/disabled; and looking after home)). Education (7 levels, summarised here as: degree level (level 1), higher education (level 2), and secondary or lower (levels 3–7)). Sending e-mails = sending and receiving e-mails; Making video calls = making video or voice calls; Finding health-r. = finding health-related information; Shopping = shopping and buying goods and services; Social networking = using social networking sites; Reading news = reading news, newspapers, and blog websites; Streaming = streaming TV, videos, radio, and listening to music; Info on Government = getting information about Government services.

## Data Availability

Data are publicly available at https://beta.ukdataservice.ac.uk/datacatalogue/series/series?id=200011. The data were accessed under project number 206540 in Jan 2021.
